# Antifungal resistance patterns of *Candida* species isolated from a private laboratory in Zambia: implications for antifungal stewardship programs

**DOI:** 10.1093/jacamr/dlag228

**Published:** 2026-10-14

**Authors:** Misheck Chileshe, Thresa Mulenga, Mwape Kunda, Steward Mudenda, Victor Daka, Vernon Julius

**Affiliations:** Mary Begg Health Services, 56 Chintu Avenue, Northrise, Ndola, Zambia; Mary Begg Health Services, 56 Chintu Avenue, Northrise, Ndola, Zambia; Mary Begg Health Services, 56 Chintu Avenue, Northrise, Ndola, Zambia; Department of Pharmacy, School of Health Sciences, University of Zambia, Lusaka, Zambia; Education and Continuous Professional Development Committee, Pharmaceutical Society of Zambia, Lusaka, Zambia; Public Health Department, School of Medicine, Copperbelt University, Ndola, Zambia; Mary Begg Health Services, 56 Chintu Avenue, Northrise, Ndola, Zambia

## Abstract

**Background:**

Fungal infections pose a significant but often underestimated public health threat. These infections range from superficial conditions, such as skin and nail infections, to invasive diseases like candidemia, which can be life-threatening. *Candida* species, in particular, have shown increasing resistance, contributing to higher morbidity and mortality among affected patients. This study investigated the resistance patterns of *Candida* species isolated from Mary Begg facilities.

**Methods:**

This cross-sectional study utilized microbiology reports containing *Candida* species and the antifungal susceptibility results from October 2024 to October 2025.

**Results:**

Of the 57 *Candida* species isolated between October 2024 and October 2025, the majority were *Candida albicans* (75.4%; 43/57) and were mainly from high vaginal swab samples (84.2%; 48/57). Among isolated *C. albicans*, resistance to fluconazole was 7.5% and to micafungin 7.1%. *Candida krusei* showed varied resistance to available drugs, with 25% of isolated species resistant to micafungin, caspofungin, and voriconazole.

**Conclusion:**

*Candida albicans* remains most prevalent and largely susceptible to first-line agents; emerging non-albicans species present a severe therapeutic challenge. Therefore, the country needs to strengthen surveillance and diagnostic capacity to address the growing problem of fungal infections and antifungal resistance.

## Introduction

Fungal infections pose a significant but often underestimated public health threat. An estimated 1.5 to 5 million fungal species exist worldwide, with over 200 known to cause infections in humans.^1^ These infections range from superficial conditions, such as skin and nail infections, to invasive diseases, such as candidemia, which can be life-threatening.^2,3^ Invasive fungal infections are the third most common cause of sepsis in hospitalized patients and are associated with high morbidity and mortality, especially among immunocompromised individuals and patients who have undergone haematopoietic stem cell transplantation (HSCT).^3^  *Candida albicans* remains the leading cause of candidemia, especially among immunocompromised individuals, including those living with HIV, cancer patients undergoing chemotherapy, and others with weakened immune systems.^4^ Without timely diagnosis and targeted treatment, these infections can cause severe complications and poor outcomes.

Antimicrobial resistance (AMR) has been recognized as a complex global health threat, and concerted efforts have been implemented to mitigate its effects.^5^ These efforts include funding research activities, improving diagnostics and surveillance, and increasing public awareness.^5^ However, most of these efforts have concentrated on antibiotic resistance and have barely addressed antifungal resistance (AFR), despite the limited number of antifungals and the presence of both acquired and intrinsic resistance (e.g. *Candida krusei* is intrinsically resistant to fluconazole).^6,7^ The increasing resistance of fungi to antifungal agents is a major concern, mainly due to misuse and improper use of antifungals, as well as to empirical treatment without proper prescription.^6^  *Candida* species, in particular, have shown rising resistance, leading to higher morbidity and mortality among affected patients.^8^

In Zambia, little is currently known about the distribution of *Candida* species and their antifungal susceptibility patterns, as fungal identification and susceptibility testing are rarely part of routine testing in many microbiology laboratories in the country.^8^

Recognizing the public health importance of fungal infections and antifungal susceptibility profiles, automated fungal (yeast) identification and minimum inhibitory concentration (MIC) testing are performed at Mary Begg Health Services (MBHS). Antifungal susceptibility results are interpreted according to Clinical and Laboratory Standards Institute (CLSI) guidelines.^9^ This study therefore aimed at investigating the resistance patterns of *Candida* species isolated within Mary Begg facilities.

## Materials and methods

### Study design and site

This was a retrospective cross-sectional study which reviewed microbiology reports for *Candida* species reported between October 2024 and October 2025. The study site was the Ba Mingilo Microbiology Laboratory, MBHS, which receives specimens from Ndola in Copperbelt Province and from Solwezi and Kalumbila in Northwestern Province.

### Sample size estimation

The study included all routine clinical samples that met the selection criteria and in which *Candida* species were isolated.

### Selection criteria

We included only reports with isolated *Candida* species. Reports also needed to include a complete clinical diagnosis and complete demographic information, such as age and sex. During the reporting period, we did not receive any specimens from patients under 18 years. Conversely, duplicate patient isolates from the same patient within 30 days were excluded. We excluded yeasts with identification confidence below 0.9 (90%) on the AutoMic-i600 analyser.

### Fungal identification and susceptibility testing

Samples were inoculated on culture plates (High vaginal swab on Blood, Chocolate, Sabouraud Dextrose Agar; urine on cystine–lactose–electrolyte-deficient agar (CLED) and Blood agar; sputum on Blood, Chocolate, MacConkey agar) and incubated for 24 hours. Plates were then read, and Gram stain performed to confirm yeasts. A fungal suspension was prepared to 2.8–3.2 McFarland standard using a Nephelometer (Autobio, China). Three microliters of suspension were added to an ID broth vial, and a drop of indicator was added to the Antimicrobial susceptibility testing (AST) broth. The ID vial, AST vial, and plate were loaded onto AutoMic-i600 for automatic identification and susceptibility testing based on colorimetric and turbidimetric detection (Autobio, China). *Candida albicans* American Type Culture Collection (ATCC) 90028 and *C. krusei* ATCC 6258 were used for Quality Control.

### Data analysis

Data were analysed using Microsoft Excel to generate susceptibility profiles.

### Ethical approval

An ethical waiver was obtained from the National Health Research and Training Institute (NHRTI—REC) (FWA:00003729; TDREC022/01/2025).

## Results

Of the 57 Candida species isolated between October 2024 and October 2025, the majority were *C. albicans* (75.4%; 43/57). Almost all isolates were from female patients (98.2%; 56/57), and most were from patients aged 30–39 years (56.1%; 32/57), as shown in Table 1. More than three-quarters of the specimens from which these yeasts were isolated were high vaginal swabs (HVS), at 84.2% (48/57). The majority of the isolates were from HIV negative patients, 82.5% (47/57). Further, almost half of the patients were pregnant, 49.1% (28/57).

**Table 1.** Distribution of *Candida* species isolated at MBHS between October 2024 and October 2025 (*n* = 57)

Parameter	*Candida albicans n* = 43	*Candida krusei n* = 8	*Candida glabrata n* = 6	Total (%) *n* = 57
Sex				
Female	42 (73.7)	8 (14.0)	6 (10.5)	56 (98.2)
Male	1 (1.8)	0 (0.0)	0 (0.0)	1 (1.8)
Age category				
18–29 years	13 (22.8)	2 (3.5)	3(5.3)	18 (31.6)
30–39 years	25 (43.9)	5 (8.7)	2 (3.5)	32 (56.1)
40+ years	5 (8.7)	1 (1.8)	1 (1.8)	7 (12.3)
Specimen type				
High vaginal swab	36 (63.2)	8 (14.0)	4 (7.0)	48 (84.2)
Urine	5 (8.7)	0 (0.0)	2 (3.5)	7 (12.3)
Sputum	2 (3.5)	0 (0.0)	0 (0.0)	2 (3.5)
HIV status				
Positive	2 (3.5)	1 (1.8)	0 (0.0)	3 (5.3)
Negative	37 (64.9)	5 (8.7)	5 (8.7)	47 (82.5)
Unknown	4 (7.0)	2 (3.5)	1 (1.8)	7 (12.3)
Medical history				
Pregnancy	22 (38.6)	3 (5.3)	3 (5.3)	28 (49.1)
Diabetes mellitus	0 (0.0)	1 (1.8)	0 (0.0)	1 (1.8)
Menorrhagia	1 (1.8)	0 (0.0)	0 (0.0)	1 (1.8)
PID^a^	1 (1.8)	0 (0.0)	0 (0.0)	1 (1.8)
Pneumonia	0 (0.0)	0 (0.0)	1 (1.8)	1 (1.8)
Pregnancy and TB^a^	1 (1.8)	0 (0.0)	0 (0.0)	1 (1.8)
Recurrent UTI^a^	1 (1.8)	0 (0.0)	0 (0.0)	1 (1.8)
Stroke	0 (0.0)	1 (1.8)	0 (0.0)	1 (1.8)
Recurrent vaginitis	2 (3.5)	0 (0.0)	0 (0.0)	2 (3.5)
None	15 (26.3)	3 (5.3)	2 (3.5)	20 (35.1)

Note: all percentages in parentheses are calculated with 57 as the denominator.

^a^PID, pelvic inflammatory disease; TB, tuberculosis; UTI, urinary tract infection.

Among isolated *C. albicans*, resistance to fluconazole was 7.5% and to micafungin 7.1% (Figure 1a). *Candida krusei* (Figure 1b) also showed varied resistance to available drugs, with 25% of isolates (same isolates) resistant to micafungin, caspofungin, and voriconazole. *Candida glabrata* showed 100% susceptibility-dose-dependent (SDD) against fluconazole (Figure 1c).

**Figure 1.**
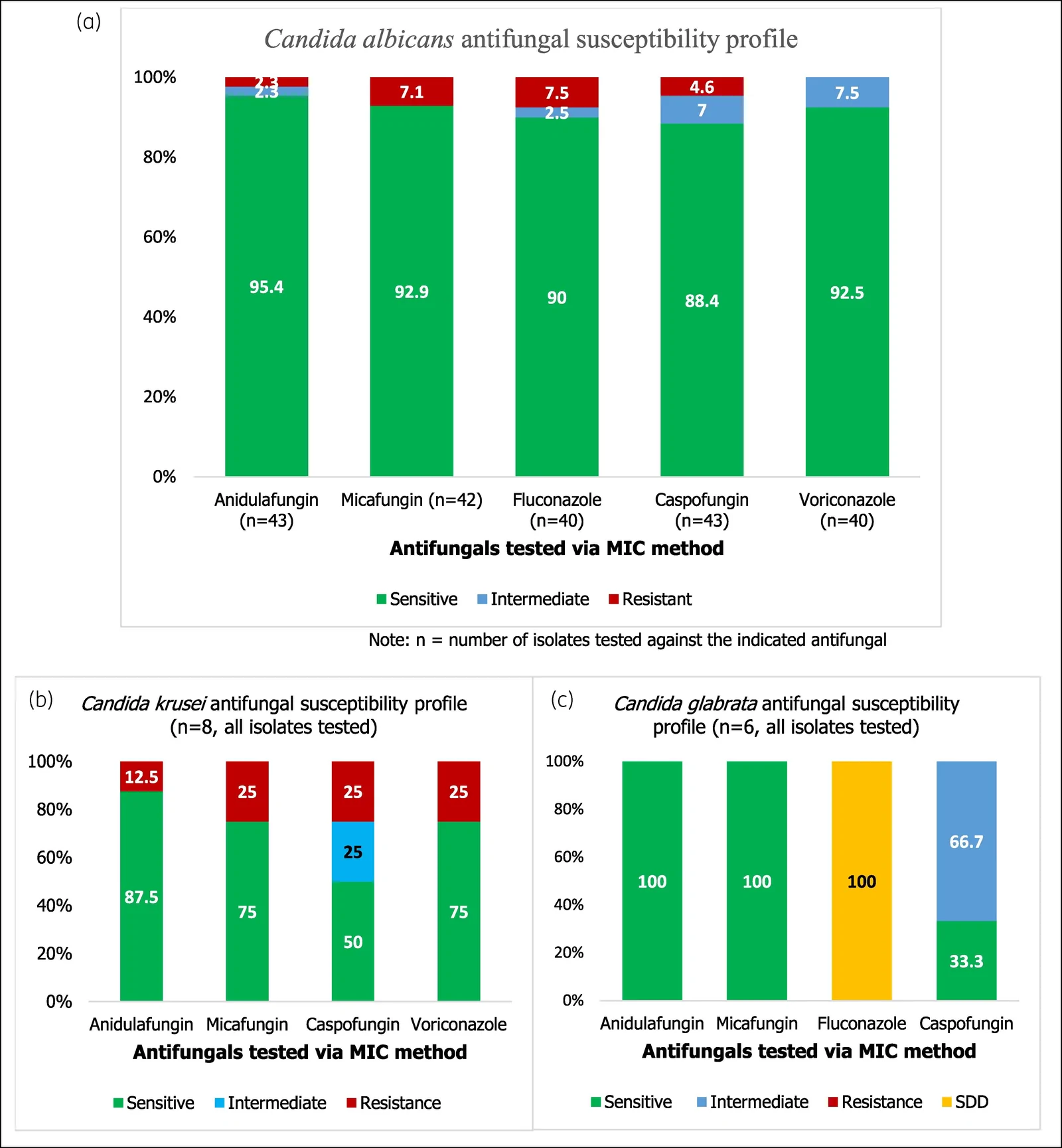
Antifungal susceptibility profile of Isolated *Candida* species.

## Discussion

This brief report aimed to identify *Candida* species and their susceptibility profile from routine clinical specimens. The clinical origin of the isolates in this report skews heavily towards the female reproductive tract, with 84.2% of *Candida* species recovered from HVS, followed by 12.3% from urine and 3.5% from sputum. This distribution strongly mirrors recent West African surveillance data.^10^ Similar to our results, they reported *C. albicans* as the primary pathogen but warned of a steep regional increase in non-albicans species exhibiting heightened resistance to widely deployed first-line azoles.^10,11^

Framing our resistance metrics within this proportion of HVS samples yields profound epidemiological insights. Firstly, because oral fluconazole is the standard, first-line empiric treatment for vulvovaginal candidiasis (VVC),^12,13^ a 7.5% fluconazole resistance barrier in a mostly vaginally derived cohort indicates an escalating need for routine species identification in recurrent cases, rather than repeated empirical dosing. Secondly, the striking 25% multidrug resistance (MDR) pattern in *C. krusei*, in which the same isolates (isolates 5 and 6) were resistant to the same antifungals (micafungin, caspofungin, and voriconazole), becomes a serious clinical roadblock in gynaecological settings. While *C. krusei* is historically a minor VVC isolate, it is heavily over-represented in chronic, recurrent vaginal infections due to its complete innate resistance to fluconazole.^14,15^ Finding that one-quarter of these isolates have also developed resistance to voriconazole and first-line echinocandins means standard therapeutic pathways fail completely. This forces reliance on topical agents (like boric acid or nystatin) or highly toxic systemic choices, vastly escalating treatment complexity.^13,14^

Although the sample is predominantly HVS, the data show that *C. albicans* remains relatively susceptible to first-line antifungal agents. Globally and regionally, *C. albicans* remains the most frequently isolated pathogen in candidiasis, and primary resistance to both azoles and echinocandins remains low.^10,15,16^ Several studies report that, in *C. albicans*, fluconazole resistance is typically an acquired trait driven by selective pressure from prolonged or empirical drug exposure, unlike echinocandins, for which resistance is correlated with mutations in the FKS1 gene.^16–18^ This emphasizes the need for fungal species identification and susceptibility testing to ensure targeted treatment, which would help in fighting this emerging pandemic.

The inclusion of *Candida glabrata* in this report highlights the unique clinical management challenges associated with non-albicans *Candida* species. Notably, the isolates demonstrate 100% susceptibility-dose-dependent (SDD) status to fluconazole, aligning with current CLSI guidelines, which do not recognize a true ‘susceptible’ category for *C. glabrata* because of the species’ intrinsically low susceptibility to azole drugs.^9^ Concurrently, the high rate of caspofungin-intermediate isolates requires careful methodological scrutiny. While an intermediate rate of 66.7% could suggest early-stage class resistance, caspofungin shows significant *in vitro* interlaboratory MIC variability, which can artificially misclassify many wild-type *C. glabrata* isolates as intermediate or resistant.^19^ This might be the case in this study, especially since the isolated *C. glabrata* was 100% susceptible to anidulafungin and micafungin.

This report offers insight into AFR and local challenges. To address fungal infections and resistance, national actions are needed. First, the country needs to collect local data on fungal pathogen distribution and resistance to guide decisions and raise awareness of the threat. This requires clinical labs to be equipped for pathogen identification and susceptibility testing. Second, the country needs to fund local research and surveillance. This will help prioritize and focus on fungal pathogens and their resistance profiles. This, in turn, will help the country to follow through with the WHO fungal priority pathogens strategic guidelines to address antifungal resistance.^20^ Thirdly, it is important to educate the public about AFR, its contributing factors, and its impact. Several studies, including local research, indicate that a lack of knowledge about antimicrobial resistance is a key driver of resistance.^6^ This is particularly evident when people self-prescribe or obtain antifungal medication without a prescription. In addition to public education, laws should be enforced to prohibit the purchase of prescription-only medicines without a prescription.

### Study limitations

This study offers insights into local antifungal susceptibility but has limitations. Small sample sizes for non-albicans species—eight *C. krusei* and six *C. glabrata* isolates—may skew resistance rates. The study also lacked molecular sequencing, limiting understanding of resistance mechanisms. Additionally, the tested antifungals may not fully reflect those available in Zambia, which includes Amphotericin B (both deoxycholate and liposomal formulations), Fluconazole, Ketoconazole, Nystatin, Griseofulvin, Terbinafine, and Itraconazole. This limitation in tested antifungals reflects the limited number of antifungals with established CLSI breakpoints. For broader applicability, future surveillance should use larger, multicentre samples and include genotypic verification.

## Conclusion

This study demonstrates a stark divergence in antifungal resistance profiles among *Candida* species isolated primarily from localized clinical specimens, dominated by high vaginal swabs. While the primary pathogen, *C. albicans*, remains largely susceptible to first-line agents, emerging non-albicans species present a severe therapeutic challenge. Most critically, the alarming 25% multidrug resistance rate in *C. krusei* to voriconazole, micafungin, and caspofungin reveals a profound clinical vulnerability, compounded by its innate resistance to fluconazole. Taken together, these findings strongly argue against the continued reliance on empirical, unguided antifungal therapy—even within community-dominated specimen pools like HVS—and underscore the immediate need for routine, species-level identification and susceptibility profiling to combat AFR. Therefore, Zambia urgently needs to establish and strengthen antifungal stewardship programs in both public and private health institutions.

## Data Availability

The individual-level dataset generated and analysed during the current study is not publicly available because it contains potentially identifiable patient-level clinical information and is subject to institutional and ethical restrictions. A de-identified minimal dataset and/or relevant aggregate data may be made available from the corresponding author upon reasonable request.
